# Advances in IL-7 Research on Tumour Therapy

**DOI:** 10.3390/ph17040415

**Published:** 2024-03-25

**Authors:** Chunxue Fu, Xinqiang Zhang, Xinyu Zhang, Dan Wang, Shuxin Han, Zhenghai Ma

**Affiliations:** Xinjiang Key Laboratory of Biological Resources and Genetic Engineering, College of Life Science and Technology, Xinjiang University, Urumqi 830046, China; 107552100988@stu.xju.edu.cn (C.F.); 107552000933@stu.xju.edu.cn (X.Z.); 107552101023@stu.xju.edu.cn (X.Z.); 107552100942@stu.xju.edu.cn (D.W.); hansx@xju.edu.cn (S.H.)

**Keywords:** IL-7, anti-tumour mechanisms, tumour therapy

## Abstract

Interleukin-7 (IL-7) is a versatile cytokine that plays a crucial role in regulating the immune system’s homeostasis. It is involved in the development, proliferation, and differentiation of B and T cells, as well as being essential for the differentiation and survival of naïve T cells and the production and maintenance of memory T cells. Given its potent biological functions, IL-7 is considered to have the potential to be widely used in the field of anti-tumour immunotherapy. Notably, IL-7 can improve the tumour microenvironment by promoting the development of Th17 cells, which can in turn promote the recruitment of effector T cells and NK cells. In addition, IL-7 can also down-regulate the expression of tumour growth factor-β and inhibit immunosuppression to promote anti-tumour efficacy, suggesting potential clinical applications for anti-tumour immunotherapy. This review aims to discuss the origin of IL-7 and its receptor IL-7R, its anti-tumour mechanism, and the recent advances in the application of IL-7 in tumour therapy.

## 1. Introduction

### 1.1. IL-7

IL-7, as a cellular inflammatory factor of the chemokine family, was first identified by Hunt et al. in 1987 while studying the potential role of bone marrow stromal cells in the development of pre-B cell subpopulations [1]. The human IL-7 gene, which is located on chromosome 8, spans 534 bp and comprises six exons and five introns. It encodes a protein consisting of 177 amino acids with a molecular weight of about 20 kDa [2]. The molecule contains three glycosylation sites, and depending on the degree of glycosylation, three protein bands may appear at approximately 20, 25 and 28 kDa when protein levels are assayed in vitro. The active form of IL-7 encodes a 25 kDa single-chain glycoprotein that forms a structure containing four alpha helices with a hydrophobic core [3]. IL-7 is mainly secreted by the bone marrow, thymus, and lymph nodes. Additionally, it is detected in sublymphatic organs such as the spleen, lymph nodes, and tonsils, as well as in non-lymphatic sites including the intestines, kidneys, lungs, skin, liver, brain, ovaries, and prostate [4,5]. This wide distribution indicates the significance of IL-7 in various physiological processes. These include its important role in B cell development, memory T cell development, the proliferation and survival of naïve T cells, and T cell development in the thymus [6]. Overall, IL-7 has a pervasive presence and diverse functions within the body.

### 1.2. IL-7R

The IL-7 receptor (IL-7R) is a heterodimeric complex located on chromosome 5 [4]. It is 1380 bp long, containing eight exons and seven introns, and encodes 459 amino acids with a molecular weight of approximately 49.5 kDa [7]. IL-7Rα is expressed in hematopoietic cells, particularly lymphoid lineages such as foetal NKs, dendritic precursors, mature T cells, and macrophages, as well as in developing T cells and B cells. There are two forms of IL-7R, membrane-bound and soluble IL-7R, each with distinct biological functions [8]. Membrane-bound IL-7R mediates IL-7 signalling, while soluble IL-7R provides regulatory control, amplifying IL-7 signalling and enhancing autoimmunity. IL-7 has been reported to play a critical role in lymphocyte maturation and differentiation through its interaction with its receptor IL-7R.

This paper reviews the origin of IL-7 and its receptor IL-7R, as well as their anti-tumour mechanisms in immunity and oncology. Additionally, recent advances in IL-7 in cancer immunotherapy and its potential for therapeutic applications are summarised and discussed.

### 1.3. Methods

#### 1.3.1. Search Strategy

Our search method was designed according to the Preferred Reporting Items for Systematic Evaluation and Meta-Analyses 2020 (PRISMA 2020) guidelines [9]. The search method retrieved all studies published between 1990 and 2024. The search strategy included (but was not limited to) the following terms: cytokines, IL-7, cancer therapy, tumours, malignant stroma, clinical data, and combination therapy. The initial search was conducted using the following tools: PubMed and Web of Science.

#### 1.3.2. Inclusion and Exclusion Criteria

Initially, 1051 relevant studies were retrieved from the databases, excluding duplicated data and irrelevant articles and accepting articles that included biological properties of IL-7 and articles related to cancer.

#### 1.3.3. Study Selection and Quality Assessment

**Figure 1:**
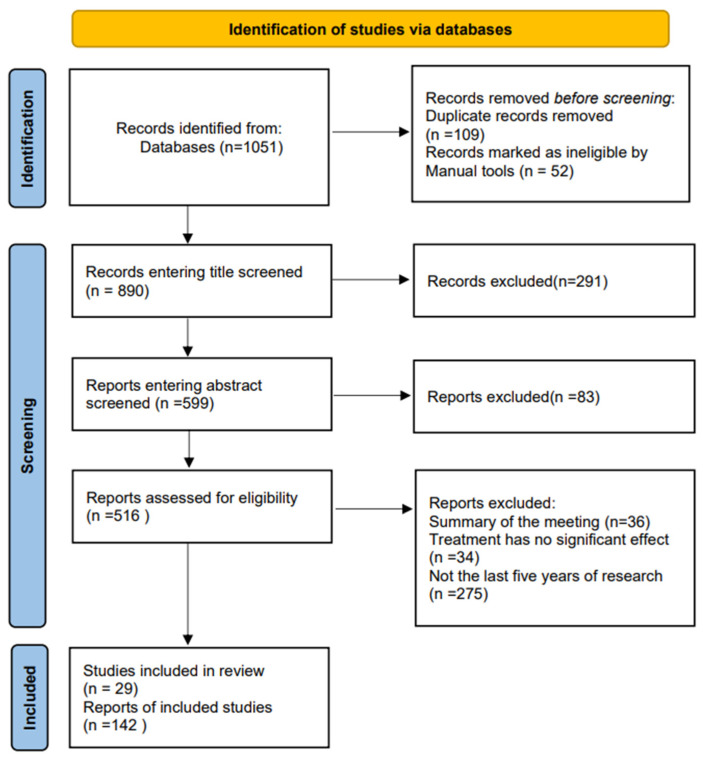
Literature screening flow chart. Initially, a total of 1051 relevant studies were retrieved from the database, 109 duplicates and 52 irrelevant articles were excluded, and 890 articles were accepted, including those on the biological properties of IL-7 and its relevance to cancer. The titles were reviewed, 291 articles were excluded, 83 articles were then excluded from the data based on the abstracts, and finally, 345 articles were excluded based on content, therapeutic efficacy, and year of publication. This process resulted in a total of 29 eligible reviews and 142 studies with their data screened.

The titles of these publications were first reviewed to exclude articles with high expressions of IL-7 in tumours and the promotion of tumour growth. Then, the abstracts of the articles were used to identify suitable articles to exclude data on the high expression of IL-7 during treatment with other drugs. Subsequently, the selected manuscripts were proofread. Finally, conference abstracts, articles with no significant effect of treatment, and articles that were not studies from the last five years were excluded (Figure 1).

#### 1.3.4. Data Extraction and Analysis

All relevant information was obtained from eligible studies. Statistical analyses were not performed due to the great heterogeneity of the included studies in terms of study design and data reporting. Therefore, the findings were presented, highlighting the similarities and differences, as well as the strengths and weaknesses, of the existing literature. Finally, this systematic evaluation did not require ethical approval.

## 2. Biological Functions of IL-7

**Figure 2:**
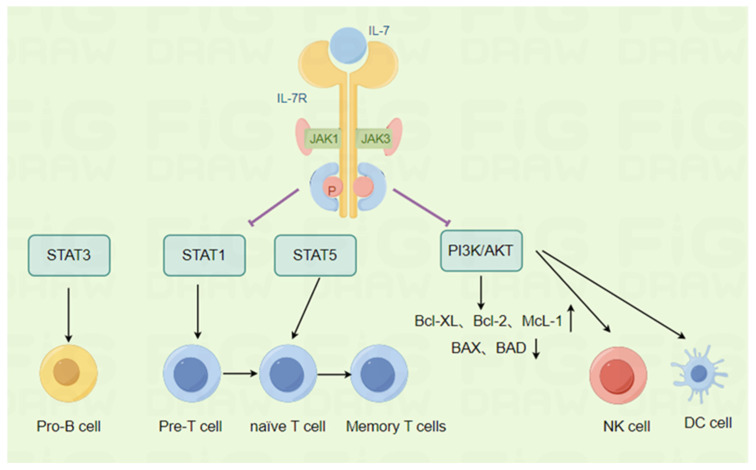
Biological functions of IL-7. The associated image materials were prepared by Figdraw 2.0. IL-7 promotes the proliferation and survival of B cell precursors by activating STAT3. Similarly, IL-7 plays an important role in the proliferation, differentiation, and survival of T cells by mediating the activation of STAT1 and STAT5. In addition to lymphocytes, IL-7 also plays an important regulatory role in the immune response of natural killer (NK) cells and dendritic cells (DC). Thus, IL-7 has a significant impact on various immune cell types.

IL-7 is a crucial regulatory factor in the development of the entire immune system, serving as a mitogen and trophic, survival, and differentiation factor for various immune cells (Figure 2), especially lymphocytes, where IL-7 is essential for B cell and T cell development, proliferation, and survival. The biological activities of IL-7 in lymphocytes are summarised in Table 1.

### 2.1. Promotion of Pre-B Cell Growth

When binding to its receptor, IL-7 activates intracytoplasmic JAK1 and JAK3 to phosphorylate STAT proteins, and the type of STAT protein determines the specificity of its response. IL-7 promotes B cell progenitor cell proliferation and survival by activating STAT3 [10]. IL-7 fails to promote the growth of mature B cells in the spleen and lymph nodes, suggesting that IL-7 can promote pre-B cell growth and has no effect on mature B cells [11]. In addition, human pre-B cells require IL-7 for direct contact with bone marrow stromal cells, which in turn activate STAT-5 for proliferation through IL-7 [12], and IL-7 is required for adult bone marrow to produce B cells [13].

### 2.2. Promote T cell Growth and Development

IL-7 plays an important role in T cell proliferation [14], differentiation, and survival by activating STAT1 and STAT5 [15].

#### 2.2.1. Promotion of Pre-T Cell Growth and Development

T cell development in the thymus begins at the double-negative (DN; CD4(-)/CD8(-)) stage and comprises four subpopulations (DN1–4) [16]. IL-7 promotes the growth of both foetal and adult thymic double-negative (CD4(-)/CD8(-)) immature T cells [17], suggesting that IL-7 may act directly as a growth factor for this cell population [18]. Furthermore, IL-7 stimulates the Bcl-2-mediated survival of DN2 and DN3 thymocytes and facilitates the differentiation of DN3 and DN4 thymocytes [5,19]. IL-7 signalling is also required for the transition from the DN to the DP phase [20]. When thymocytes reach the DP phase, they differentiate into single CD4+ or CD8+ T cells, at which point IL-7 is also required for cell survival [21].

#### 2.2.2. Promotion of T Cell Proliferation

IL-7 plays a crucial role in thymocyte development and also serves as a vital cytokine in T cell development and proliferation, maintaining immune homeostasis, and ensuring normal T cell function. JAK3 can induce p85 phosphorylation and activate the PI3K/Akt pathway [22]. When IL-7/IL-7R stimulates JAK1/3, the p85 subunit of PI3K becomes phosphorylated, leading to Akt kinase activation and the subsequent stimulation of the PI3K/Akt pathway. This activation results in the upregulation of anti-apoptotic proteins Bcl-XL, Bcl-2, and Mcl-1 of the Bcl-2 protein family, along with the downregulation of pro-apoptotic proteins BAX and BAD, ultimately promoting cell survival, proliferation, and growth [23]. In mice, IL-7 mediates T cell proliferation and activation, a process that is subdued by PI3K inhibitors [24]. This implies that the PI3K/AKT pathway plays a crucial role in IL-7 signalling in the cell cycle. Research has shown that using IL-7 in mice results in a preferential increase in the number of CD8+ cells over CD4+ cells and enhances the proliferation of both CD4+ and CD8+ T cells [25]. Moreover, IL-7 fosters the survival and homeostatic proliferation of both CD4+ and CD8+ T cells after peripheral blood withdrawal [26]. IL-7 also promotes the proliferation of activated CD4+ and CD8+ peripheral blood T cells [27]. IL-7 can help T cells restore homeostasis through the signal transducer suppressors of the cytokine signaling proteins (SOCS) pathway and STAT5, a cytokine signalling inhibitor [28]. In addition, the JAK/STAT pathway is not only activated but can also be inhibited by the family of SOCS, creating a negative feedback loop [29].

The PI3K pathway plays a critical role in promoting cell viability and the cell cycle by phosphorylating AKT. This process also phosphorylates downstream targets including GSK-3, FOXO-1, and FOXO-3a, subsequently upregulating the expression of BCL-2 and downregulating the cell cycle inhibitor p27kip1. Multiple pieces of evidence support the idea that PI3K/AKT is a significant signalling pathway for IL-7R activation, which ultimately leads to the upregulation of the GLUT-1 transporter and glucose uptake. This, in turn, enhances peripheral T cell metabolism, homeostasis, and survival.

#### 2.2.3. Maintenance of Memory T Cells

IL-7 is critical not only for T cell development but also for the survival of naïve T cells and the formation and maintenance of memory T cells [29]. In addition, IL-7 signaling helps to maintain CD8+ memory T cells by activating STAT5 and STAT3 [30]. IL-7 also plays a role in regulating the number of memory CD8+ T cells produced [31] and in guiding naïve CD8+ cells to transition into memory CD8+ T cells [32], which promotes the proliferation [33] and activity of effector T cells. Furthermore, IL-7 has a significant impact on the transition from effector to memory T cells [34]. In CD8+ memory T cells, IL-7 can act both directly [33] and indirectly by stimulating IL-2 production and IL-2 receptor expressions [35]. Importantly, in the presence of lymphopenia, the survival and proliferation of naïve T cells are reliant on IL-7, as indicated by the inability of these cells to survive in hosts lacking IL-7 [36]. Likewise, in cases of lymphopenia, the survival and maintenance of CD4+ memory T cells depend on IL-7 and the TCR pathway [37]. Likewise, a study on a mouse model of airway inflammation shows that allergen-specific memory CD4+ T cells rely on IL-7 for their survival in the lungs and airways, as well as for their homeostatic proliferation in lymph nodes [38]. Moreover, reducing IL-7 by injecting IL-7 antibodies in normal mice leads to a significant decrease in the number of naïve T cells [39]. Additionally, IL-7 also affects the cytotoxicity and drug resistance of T cells [40].

#### 2.2.4. Treg Cell Development

IL-7 is essential for the development, homeostasis, and function of regulatory T cells (Tregs) [41]. However, there have been conflicting results regarding the role of IL-7/IL-7R in Treg development. While IL-2 is known to play a crucial role in Treg homeostasis [42], it has been discovered that CD25-deficient Tregs rely on IL-7 for survival in the absence of IL-2 [43]. On the other hand, some studies have indicated that IL-7Rα alone is not enough to promote mature Treg development [44]. Although necessary for Treg development [45], IL-7Rα has a notably low expression in mature Tregs [45]. Consequently, further research is needed to fully understand the role of IL-7 in Treg development.

**Table 1:** Biological activities of IL-7 in lymphoid cells. Reproduced with permission from Ref. [5]. Copyright 2022, Published by Elsevier Ltd.

Function	Cell Types	Activities
Development	CLP	“Transcription of Genes Related to the B Cell Lineage” [46]
	Thymocytes	Recombination at the TCR γ,β loci for VDJ [47]
	Thymocytes	Preventing premature TCR α chain recombination during the selection of the β chain [48]
	Double positive thymocytes	Silencing CD4 transcription and activating CD8 transcription leads to the conversion of cells to a single positive state [49]
	B cell progenitors	Recombination of V segments in heavy-chain locus [7]
	B cell progenitors	Downregulation of IL-7 receptor expression and completion of Ig light gene rearrangement [50]
Survival	Thymocytes (DN cells)	Stimulation of Bcl2 expression [51]
	B cell progenitors	Activation of Jak3/Stat5-dependent pathways [52]
	Naïve T cells	Expression of pro-survival Bcl2 family
	Memory T cells	members in mitochondria [53]
	NK cells	Increased expression of Bcl2 [54]
Proliferation	B cell progenitors	Jak3/STAT5 [55]
	Thymocytes	Cooperation between IL-7 and Notch signals [56]
	Activated T cells	Increase in the production of IL-2 and expression of IL-2 receptors [57]
Activation	CD8 Effector cells	Expansion of CD8+ cells and increased cytolytic activity [58]
	Memory T cells	Cytokine production [59]
Differentiation	Transition of mature effector T cells to memory cells	The hypothesis of a combination of survival signals with epigenetic modifications has not yet been fully defined [60]
Homeostatic proliferation	Naïve T cells	The downregulation of p27 cyclin-dependent kinase inhibitor activity leads to increased mTor phosphorylation [61]
	NKT cells	Still undefined
	Memory T cells	Still undefined

### 2.3. Promotion of Lymphangiogenesis

IL-7 plays a crucial role in the lymphocytes and also has a significant impact on the lymphovascular system. IL-7 interfering with the activation of GSK3 through AKT causes the upregulation of NFAT2 activity [62]. Another pathway involving the JAK1/STAT5 pathway or the phosphorylation of JAK3 can activate NFAT2, which targets a wide range of cytokines and growth factors with pro-inflammatory and angiogenic activities [29]. IL-7 is also responsible for activating lymphatic endothelial cells (LECs), a process that promotes cutaneous lymphatic drainage [63]. IL-7 also promotes lymphatic vessel dilatation during inflammation or post-immunisation lymphatic dilatation [64]. IL-7 mediates the formation of lymphoid organs by increasing the number of lymphoid tissue inducer (LTi) cells, a process known as lymphangiogenesis [65]. S. Chappaz et al. [66] showed that lymph node development was defective in IL-7-deficient mice and that the IL-7 expression increased the number of LTi cells and restored lymph node development.

### 2.4. Other Effects

IL-7 has been reported to play an important regulatory role in the immune response of natural killer (NK) cells and dendritic cells (DC). Furthermore, IL-7 preferentially induces the JAK-mediated activation of STAT5 [10], which is involved in the transduction of the pro-survival, mitogenic, trophic, and differentiation signals of immune cells [29]. Additionally, IL-7 maintains the homeostasis and normal function of mature T cells and promotes the survival of mature NK cells [67]. Consequently, IL-7 is essential for the development of NK cells.

## 3. Anti-Tumour Mechanism

**Figure 3:**
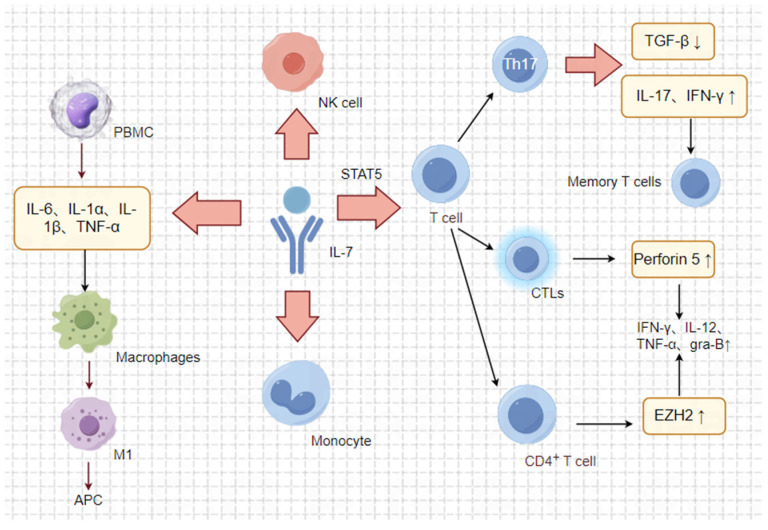
Anti-tumour mechanism of IL-7. The associated image materials were prepared using Figdraw 2.0. IL-7 plays a critical role in the tumour microenvironment by attracting T lymphocytes and promoting their infiltration into tumours, thus exerting an anti-tumour effect. Moreover, IL-7 also enhances the cytotoxicity of cytotoxic T lymphocytes (CTLs) and induces a degree of lymphokine-activated killer (LAK) activity. Additionally, IL-7 acts as a cytotoxic agent by modulating the release of cytokines, such as IFN-γ, IL-1β, IL-1α, and TNF-α, by immune cells. This modulation promotes macrophage polarisation toward the M1 phenotype and the expression of a pro-inflammatory milieu, which, in turn, promotes the differentiation, maturation, and effective activation of antigen-presenting cells (APCs). Overall, IL-7 serves as a key regulator in the immune response within the tumour microenvironment, influencing various immune cell activities and further promoting the anti-tumour environment.

Due to its crucial role in the growth and development of various immune cells, IL-7 holds great promise for cancer therapy in a clinical setting. The specific anti-tumour mechanism of IL-7 is illustrated in Figure 3.

### 3.1. Enhancing Adaptive Immunity

IL-7 plays a crucial and multifaceted role in the tumour microenvironment, selectively influencing the development of naïve and memory T cells while avoiding the stimulation of immunosuppressive Tregs. Moreover, the cytokine IL-7 promotes the differentiation of CD4+ T cells into Th9 cells with enhanced anti-tumour activity. Specifically, the complex formed by IL-7 with IL-7R-Fc has been shown to induce an anti-tumour response by increasing the infiltration of T cells into tumours through the CXCR3 chemokine signalling pathway [68].

IL-7 promotes the development of helper T17 (Th17) cells. In the tumour microenvironment, Th17 cells enter and expand through antigen-presenting cells. Furthermore, they promote the operation and maintenance of effector T cells and NK cells by inducing primary tumour cells to produce CXCL9 and CXCL10. As a result, Th17 cells can facilitate protective anti-tumour immunity by recruiting pro-inflammatory immune effector cells. Studies have demonstrated that IL-7 not only promotes Th17 cell proliferation to enhance anti-tumour efficacy in a mouse B16F10 tumour model [69] but also is necessary for developing effector Th17 cells into resident memory T cells [70,71]. Some of these resident memory cells express IL-17, while others express IFN-γ. Thus, IL-7 is vital for the development of Th17. In a mouse glioblastoma model, Shireman JM et al. [72] demonstrated that IL-7-stimulated T cell translocation to the brain facilitates the formation of Th17 cells and generates persistent long-term anti-tumour immunity.

### 3.2. Reversal of Immunosuppression

IL-7 also enhances anti-tumour immune responses by counteracting immunosuppressive mechanisms. IL-7 promotes the differentiation of Th17 cells by inducing the expression of SMAD ubiquitination regulatory factor 2, which inhibits the TGF-β signalling pathway and deregulates the inhibition of effector T cells by Tregs. IL-7 and TGF-β act antagonistically, and the inhibition of TGF-β enhances the anti-tumour immune response, thereby inhibiting tumour growth. Tumour-infiltrating suppressor cells often express adenosine, which accumulates extracellularly and suppresses CD8+ T cells. Findings from a single study indicate that IL-7 signaling is crucial in protecting CD8+ T cells from this suppression and in preventing immunosuppression. These results may have implications for immunotherapies that target the adenosine pathway [58].

### 3.3. Enhancing Immune Cell Recruitment

IL-7 has been reported to also upregulate the expression of the adhesion molecule V-CAM on endothelial cells. This upregulation promotes the recruitment of circulating immune cells. YW Choi showed that IL-7-Fc induced cervicovaginal epithelial cells to stimulate endothelial cell V-CAM expression, resulting in the inhibition of cervicovaginal carcinoma growth in mice [68].

### 3.4. Enhancing the Expressions of Inflammatory Factors

IL-7 enhances the cytotoxicity of cytotoxic T lymphocytes (CTLs), as well as monocytes, NKT, NK, and LAK cells. Additionally, IL-7 induces a degree of lymphokine-activated killer (LAK) activity. It also promotes the secretion of perforin 5 from CTLs in a STAT5-dependent manner and stimulates the expressions of IFN-γ [58], IL-12, and MIG [58]. Furthermore, in the presence of lymphopenia, IL-7 not only leads to the IL-7-dependent activation of STAT1 and STAT5 but also enhances the T cell response to IFN by regulating STAT1 protein expression levels [73]. In response to IL-7 stimulation, human peripheral blood mononuclear cells release a variety of cytokines, such as IL-6, IL-1α, IL-1β, and TNF-α. Additionally, in the presence of IFN-γ and TNF-α, it promotes macrophage polarisation toward the M1 phenotype and creates a pro-inflammatory environment [74]. Finally, it can also confer stat5-dependent multifunctionality on CD4+ T cells, resulting in an increased expression of the histone methyltransferase EZH2, which in turn promotes the expressions of IFN-γ, IL-2, TNF-α, and granzyme B.

A pro-inflammatory environment is crucial for the differentiation, maturation, and efficient activation of antigen-presenting cells (APCs). These cells can then provide co-stimulatory signals to cytotoxic T cells. Host APCs are necessary for successful antigen presentation, even in highly immunogenic tumours. In a study on gastric cancer, IL-7 was found to be closely associated with regulatory T cells. They may influence gastric carcinogenesis and progression by regulating immune infiltration and inflammation [75].

## 4. IL-7 in Tumour Therapy

Based on the powerful immunomodulatory effects of IL-7, which can act directly or indirectly on tumour cells, IL-7 can effectively reduce the tumour load in tumour therapy [76] and exert anti-tumour effects by enhancing eradication or adaptive immunity. Table 2 provides a summary of examples of IL-7 that are currently undergoing clinical trials.

### 4.1. Targeted Therapies

It is fascinating to learn about the potential of IL-7 in immune reconstitution and its anti-tumour activity based on preclinical studies. The fact that IL-7 has been well tolerated in early clinical trials and that there have been no reports related to greater irritation responses is promising. The development of drugs targeting the IL-7 gene for autoimmune diseases and cancer seems very promising, particularly the use of recombinant IL-7 proteins, IL-7 gene vaccines, IL-7 receptor antagonists, and IL-7 signalling pathway blockers.

#### 4.1.1. IL-7 Recombinant Protein

Previous studies have shown that IL-7 has a short half-life in vivo and can act only in an autocrine or paracrine manner over short distances. Scientists have therefore extended its half-life by genetically engineering it to fuse with the c-terminal end of hIgG1 Fc to enhance IL-7 activity to increase the immune effect of IL-7 in tumour therapy. Previous clinical trials have shown that IL-7 fusion proteins are stable [77], well tolerated, have a prolonged half-life [78], and have good prospects for clinical application. YW Choi et al. [68] showed that IL-7 Fc successfully inhibited the growth of cervicovaginal tumours in mice. Clinical trials of immunotherapy with human IL-7-Fc are also underway. rhIL-7 (also known as rhIL-7-hyfc) has been developed by Genexine with an extended half-life and reduced off-target immunogenicity. In a lymphocytopenic mouse model of melanoma, IL-7-Fc treatment inhibited tumour growth by increasing the number of overtly metastatic CD8+ T cells in tumour tissue and tumour-draining lymph nodes [79]. A clinical result for the treatment of recurrent glioblastoma (GBM) showed that administering different doses of rhIL-7-hyFc to patients separately significantly increased TLC in all but one patient in a dose-dependent manner and did not result in severe toxicity [80].

NT-I7, a longer-acting IL-7 Fc, was tested for its ability to prevent systemic lymphopenia and improve survival in a GBM mouse model, and it was demonstrated that in a mouse model of glioma in situ, NT-I7 increased central and effector memory CD8+ T cells in both lymphoid organs and tumours [81]. In addition, it significantly inhibited the growth of glioma in situ and improved the survival rate in mice compared to the control group [81]. A phase I/II trial evaluating NT-I7 in patients with high-grade glioma (NCT03687957) is also ongoing [81].

#### 4.1.2. IL-7 Gene Vaccine

The IL-7 gene vaccine is a gene therapy that enhances anti-tumour immune responses by introducing the IL-7 gene into tumour cells or immune cells to cause them to produce the IL-7 protein, thereby increasing IL-7 levels. A notable clinical trial of IL-7 gene therapy in cancer treatment is the safety and feasibility in patients with relapsed or refractory haematological malignancies. Early results from this trial suggest that IL-7 gene therapy may enhance anti-tumour immune responses and improve clinical outcomes in such patients [82]. In conclusion, preclinical and early clinical data suggest the potential to enhance anti-tumour immune responses [83]. However, further studies are needed to fully understand its safety and efficacy in the treatment of cancer.

#### 4.1.3. IL-7 Receptor Antagonists and IL-7 Signalling Pathway Blockers

IL-7 plays a complex role in cancer pathology, and there has been increasing interest in its potential role in cancer cachexia. Cachexia is a state in which cancer patients suffer from systemic dysfunctions triggered by the disease or its treatment, resulting in malnutrition, reduced immune function, and a decline in quality of life [84]. Studies have shown that the effects of IL-7 on immune cell function and metabolism may contribute to cancer cachexia. Specifically, IL-7 levels are significantly elevated in cancer patients with cachexia compared to those without cachexia [85], and the promotion of differentiation and activation of T cells by IL-7 may lead to muscle wasting and weight loss. Additionally, IL-7 has been associated with dysregulated energy metabolism, leading to malnutrition in cancer patients [86]. There is also evidence that IL-7 may affect the mental state of cancer patients by influencing neurotransmitter levels, leading to psychological problems such as anxiety and depression, further reducing the patient’s quality of life [87].

The association between IL-7 and cancer cachexia highlights the potential for targeted therapies to mitigate the effects of cachexia in cancer patients. Strategies targeting IL-7, such as designing IL-7 receptor antagonists or blockers of the IL-7 signalling pathway, are being investigated to mitigate cancer cachexia. Researchers have engineered an alternative antagonist to the anti-IL-7R antibody, a 64-amino acid-less peptide based on the structure of the IL-7R antibody, which is hyperstable and blocks the IL-7-induced phosphorylation of Stat5 in cell lines [88]. This antagonist could be clinically tested in patients with relapsed paediatric acute lymphoblastic leukaemia (ALL). Clinical trials of IL-7 receptor agonists have investigated their safety, tolerability, and efficacy in a variety of cancers to identify the optimal dosage, treatment schedule, and patient population for IL-7 receptor agonist therapy [89].

IL-7-targeted therapies have made breakthroughs in several cancer types, including non-small cell lung cancer (NSCLC), lymphoma, colorectal cancer, and melanoma. Studies have shown that IL-7 can increase the number and function of tumour-infiltrating lymphocytes and improve the efficacy of immune checkpoint inhibitors such as PD-1 and PD-L1 antibodies. A clinical trial found that combination therapy with IL-7 and immune checkpoint inhibitors significantly improved survival and response rates in NSCLC patients compared to using immune checkpoint inhibitors alone [90]. IL-7 has been extensively studied in the treatment of lymphoma. One study found that IL-7 was able to increase the number of CD4+ and CD8+ T cells and improve the function of these cells in lymphoma patients. The combination of IL-7 with chemotherapy or immunotherapy has been suggested as a potential therapeutic strategy for improving the treatment response and survival in lymphoma patients [91]. IL-7 has also shown potential in the treatment of colorectal cancer. Studies have shown that IL-7 can increase the number of tumour-infiltrating lymphocytes in colorectal cancer patients and improve the activity of these cells. A clinical trial found that combining IL-7 with chemotherapy significantly improved the survival rate of patients with advanced colorectal cancer [92]. IL-7 has also shown potential efficacy in the treatment of melanoma. Studies have shown that IL-7 can increase the number of anti-tumour immune cells in melanoma patients and improve the activity of these cells. A clinical trial found that combining IL-7 with immune checkpoint inhibitors significantly improved treatment response rates and survival in patients with advanced melanoma [93].

### 4.2. Combination Therapy

#### 4.2.1. Combination with Other Cytokines

IL-7, which has demonstrated strong effectiveness in fighting tumours when used alone, can also be utilised in combination with other cytokines such as IL-12, IL-15, and GM-CSF.

IL-12 is a widely recognised pro-inflammatory cytokine that plays a crucial role in activating both NK cells and T cells, creating a connection between innate and adaptive immunity. This ultimately results in a more effective enhancement of anti-tumour immunity. Tasaki M [94] showed that in a murine lung cancer (LLC) model, combined treatment with IL-7 and IL-12 promoted the activation of CD8+ T cells, which had a better effect on CD8+ T cell activation than the control group expressing IL-12 alone. Shireman JM et al. constructed a fusion of IL-7 and GM-CSF, i.e., GIFT-7, which led to tumour regression after three weeks of administration in a mouse glioma model [72].

IL-15 is also an important immunomodulator in cancer therapy. In a recent study, the structural domains of other cytokines were genetically engineered to bind to IL-7 to form a bifunctional protein fusion. Song et al. constructed a dual cytokine: a hybrid protein of IL-7 and IL-15 and injected different doses of rIL7/IL15 at the tumour site every 2 days in a mouse CT26 tumour model. The results of the study showed that rIL7/IL15 inhibited tumour growth in a dose-dependent manner by approximately 60% at the 2.5 μg level and >80% at the 20 μg level, which are both superior to cytokine monotherapy in terms of anti-tumour effect [95]. Recently, an injectable hydrogel microsphere integrated training field (MS-ITC) containing anti-CD3 and anti-CD28 antibodies and bovine serum albumin nanoparticles encapsulated with IL-7 and IL-15 was newly developed [96]. The MS-ITC were injected into osteosarcoma tumour tissues of homozygous mice. The results of the study showed that combined treatment with IL-7 and IL-15 induced the recruitment and activation of TIL-Ts, which disrupted tumour cell growth and ultimately led to the regression of the primary osteosarcoma [96].

#### 4.2.2. Combined Anti-Cancer Drug Therapy

IL-7 has shown strong anti-tumour activity as a monotherapy and it can also be combined with other drugs to enhance anti-tumour activity.

Cisplatin (DDP) is a therapeutic agent used to treat non-small cell lung cancer (NSCLC) but has the disadvantage of making cancer cells resistant to the drug. The combination of IL-7 with DDP induces NSCLC tumour regression while reducing ABCG2 levels in tumour tissue. This result suggests that IL-7 can also overcome the multidrug resistance of DDP and restore its chemosensitivity [97]. Currently, sip-T is the only immunotherapy approved by the US Food and Drug Administration (FDA) for the treatment of metastatic desmoplasia-resistant prostate cancer (mCRPC). The results from a preclinical study showed that the combination of IL-7 and sip-T significantly inhibited tumour growth in a mouse model of prostate cancer, with potential clinical efficacy that should be further evaluated in clinical trials [98]. The results of a phase II clinical trial (NCT01881867) showed that the combination of IL-7 and sip-T in mCRPC patients resulted in a significant increase in antigen-specific humoral IgG and T cells, and increased expressions of activation markers and beneficial cytokines such as IL-2 and IFN-γ were observed. In comparison to the control group, the group receiving combined IL-7 and sip-T treatment showed decreased levels of the prostate cancer marker PSA at week 6. Additionally, there was an increase in the number of patients with PSADTs longer than 6 months.

The combination of IL-7 with antibodies has also shown promising anti-tumour effects in recent studies. Gou et al. [99] found that IL-7 in combination with oxaliplatin inhibited the growth of metastatic tumours and enhanced the anti-tumour activity of oxaliplatin in a mouse model of lung and peritoneal metastases. Combinations of IL-7 with temozolomide for the treatment of glioblastoma [64] and with atelizumab for the treatment of skin cancer [100], melanoma, and squamous cell carcinoma of the skin [101] are under ongoing investigation. A clinical trial of IL-7 and atezolizumab for the treatment of urothelial carcinoma (NCT03513952) is also ongoing, and IL-7-Fc is being used in combination with pembrolizumab in a clinical trial of triple-negative breast cancer [102].

Recent studies have identified a novel IL-7 agonist with the pharmacologically active peptide MDK1472 forming MDK-703, a complex that induces an immune cell profile in an NSG mouse model very similar to that produced by IL-7, with properties more suitable for therapeutic applications than natural IL-7. MDK-703 was administered to cynomolgus monkeys through a single dose of 1 mg/kg via intravenous, subcutaneous, or intramuscular injection. MDK-703 was well tolerated with no adverse effects, suggesting that MDK-703 may be a clinical candidate for oncological treatment [103]. A trial of MDK-703 with patients with advanced solid tumours is currently underway (NCT05716295).

#### 4.2.3. Combination of Oncolytic Viruses

Oncolytic virus therapy is an emerging approach to tumour immunotherapy. Oncolytic viruses (OVs) are specifically designed to target and destroy tumour cells, leaving healthy cells unharmed [104]. Not only can oncolytic viruses lyse cells directly, but the lysed tumour cells can also release tumour-associated antigens [4], as well as induce tumour immunity by releasing cytokines and exhibiting damage-associated molecular patterns to enhance antigen presentation and immune activation [105].

IL-7 has shown promising results in clinical trials with the combination therapy of IL-7 with lysosomal viruses used to enhance anti-tumour effects. The construction of a lysosomal adenovirus Ad5/3-E2F-d24-hIL-7 expressing IL-7 targets the tumour microenvironment for high expressions, overcoming systemic delivery problems and improving therapeutic efficacy. The effects of this recombinant virus on tumour growth, immune cell activation, and cytokine profiles in the tumour microenvironment were evaluated in three clinically relevant animal models. The results showed that Ad5/3-E2F-d24-hIL-7 promoted pro-inflammatory cytokine expression and CD4+ and CD8+ T cell activation and migration. Local treatment in hormone-treated mice significantly reduced pancreatic cancer growth [106].

Nakao et al. developed a lysogenic poxvirus co-expressing the cytokines IL-7 and IL-12 to enhance the anti-tumour effect. When this recombinant virus was treated via intratumoral administration, anti-tumour activity was shown in all three tumour models, with a 92.9% inhibition of tumour growth in the B16-F10 melanoma model and 53.3% inhibition of tumour growth in CT26. There were mice in the B16-F10 melanoma model that showed tumour regression, and mice with complete tumour regression were resistant to rechallenge with the same tumour cells; it has been suggested that the injection of the virus results in the establishment of long-term memory specific to the tumour [107].

In a recent study, several immunotherapeutic genes were inserted into the viral genome to enhance its oncolytic activity. Herpes simplex virus-2 (HSV-2) vectors are a promising type of lysogenic virus. oHSV2 co-expressing IL-7 and the chemokine CCL19 was constructed by Hu et al. [108], and its anti-tumour efficacy was tested in 4T1- and CT26-loaded mouse models. The results showed that oHSV2-IL7-CCL19 significantly inhibited tumour growth in mice compared to controls.

The above results indicate that IL-7 combined with lysosomal viruses has good tumour-killing and anti-tumour immune effects in the treatment of malignant tumours and has good prospects for clinical application.

#### 4.2.4. Combined CAR-T Cells

Chimeric antigen receptor (CAR-T) cells are engineered receptors that are added to normal T cells, allowing them to effectively target and kill tumour cells expressing specific antigens. In recent years, IL-7, in combination with CAR-T cells, has been widely used in preclinical and clinical trials for a variety of solid tumours and haematological cancers, with promising results [109,110,111,112,113,114,115,116,117].

Studies have demonstrated that IL-7 promotes the in vitro expansion of CAR-T cells [118], and IL-7 has also been shown to enhance the memory properties of CAR-T cells [119,120]. When IL-7 was combined with CAR-T cells in relapsed and refractory haematological malignancies, tumour growth was significantly inhibited [121]. A clinical trial involving IL-7 × CCL19 CAR-T cells combined with tirilizumab was conducted on 11 patients with relapsed or refractory large B cell lymphoma. The results demonstrated that the autologous 7 × 19 CAR-T cells, in combination with tirilizumab, displayed good efficacy in treating relapsed and refractory large B cell lymphoma while also causing manageable side effects [122]. After 30 days of receiving 7 × 19 CAR-T cell injections at the tumour site, a patient with advanced hepatocellular carcinoma experienced the complete disappearance of the tumour. In a separate case, a patient with advanced pancreatic cancer experienced a near-total disappearance of the tumour after 240 days of intravenous injections [109]. Wang SY et al. [123] showed that IL-7 in combination with (NICE) CD19 CAR-T cells exhibited good therapeutic efficacy in a mouse model of B cell lymphoma compared to the control group. The tumour growth rate was significantly reduced, and survival was improved in the treatment group receiving intravenous IL-7.

Due to the substantial anti-tumour activity displayed by IL-7 and CAR-T cells in haematological malignancies, their combination has been incorporated into clinical trials for diverse cancer immunotherapies. However, previous studies have shown that CAR-T calls have poor efficacy in solid tumours and T cell recruitment; survival and proliferation are important limiting factors for CAR-T cell therapy in solid tumours [124]. CAR-T cells armed with IL-7 prolong the persistence of CD4+ T cells and enhance the anti-tumour response. Therefore, Luo et al. [115] developed a CAR-T cell co-expressing the cytokines IL-7 and CCL21 (7 × 21 CAR-T). The results of the study showed that 7 × 21 CAR-T cells significantly inhibited tumour growth and even achieved tumour elimination after treatment compared with conventional CAR-T cells in the PANC02-A2 pancreatic cancer model. The co-expression of IL-7 and IL-7 Flt3L CAR-T cells improves the therapeutic efficacy of heterogeneous glioblastoma in mice [119]. A novel CAR-T cell targeting GPC3 induced the co-expression of IL-7 and high levels of CCL19. In a humanised NSG mouse xenograft model, GPC3-CAR-T killed tumour cells at twice the rate of conventional CAR-T cells, and this led to tumour regression. Therefore, a phase I clinical trial was initiated in which GPC3-7-19 CAR T cells showed good safety and anti-tumour efficacy in patients with hepatocellular carcinoma (HCC) [125].

Triple-negative breast cancer (TNBC) is a very aggressive malignancy for which there are no effective targeted therapies. To use CAR-T cells for the treatment of TNBC, the anti-tumour efficacy of CAR-T cells was enhanced with the IL-7 receptor (C7R). It was shown that C7R-enhanced CAR-T cells exhibited significant anti-tumour activity in the NOD/SCID mouse model of TNBC, with a significant reduction in tumour volume compared to the PBS group, and a prolonged survival time of CAR-T cells in mice [114]. Recombinant protein technology could be a potential approach due to its advantages of reduced toxicity and lower cost. The most recent study was a recombinant protein targeting HER-2, which is a fusion of the anti-HER-2 single-chain fragment variable structural domain CCL19 with IL-7. The results of the study showed that in a humanised NSG mouse model of gastric cancer, control tumours consistently increased in volume and 2/5 mice developed skin ulcerations localised to the burdened tumour. On the other hand, following three treatments with HER2 scFv-CCL19-IL-7, there was a marked reduction in tumour volume and consistent inhibition of tumour growth, indicating potent and sustained tumour suppression [126].

Furthermore, IL-7 has been shown to have anti-tumour effects in T cell receptor (TCR-T) cells, in addition to its known impact on CAR-T calls. TCR-modified T cells for open T cell therapy have shown promise in the treatment of metastatic melanoma and other malignancies. A common protocol for TCR-modified T cells involves activating the T cells through CD3 stimulation, allowing for the efficient transfer of tumour-responsive receptors using viral vectors [127]. It has been demonstrated that using IL-7 to generate TCR-modified T cells in the absence of CD3 activation is a feasible strategy for enhancing over-the-counter T cell therapy for melanoma and other types of cancer [127]. A study was conducted to genetically engineer TCR-T cells (7 × 19 P1A T) to express both IL-7 and CCL19, which resulted in complete tumour regression and prolonged survival in half of the mice after 7 × 19 P1A T treatment in a murine mast cell tumour P815 model [109].

Adoptive T cell therapy (ACT) has demonstrated remarkable effectiveness in combating tumours. This treatment depends on a reservoir of functional T cells capable of continually targeting and destroying tumour cells [128]. However, a persistent shortage of functional T cells has been recognised as a significant obstacle to achieving a lasting response. Equipping infused T cells with stem-like qualities to produce memory T cells with the ability to self-renew and differentiate in multiple ways, akin to pluripotent stem cells, is crucial and holds great promise for improving T cell function and sustaining anti-tumour immunity [128].

The findings above suggest that combining CAR T cell therapy with IL-7 expression could have a positive anti-tumour immune effect, making it a promising option for clinical use.

#### 4.2.5. Other

IL-7 can also be used as an adjuvant in combination with tumour vaccine therapy to increase T lymphocyte infiltration into the tumour microenvironment, thereby enhancing the anti-tumour effect of cancer vaccines. IL-7 adjuvants also help to prolong the survival of activated T cells, enhance effector responses, and increase cytokine production, thereby improving the immune effect of tumour vaccines. Current examples of IL-7 in combination with different tumour vaccines in clinical trials are summarised in Table 3. Clinical trials of rhIL-7-hyFc in combination with various tumour vaccines are also underway [98]. IL-7 promotes antibody and T cell responses to HCV DNA vaccines [129]. In addition, the same adjuvant effect of IL-7 has been shown to promote the efficacy of DC vaccines. Thus, the combination of IL-7 and tumour vaccines has great potential for use in tumour therapy.

## 5. Conclusions and Outlook

The results of a large number of preclinical and clinical studies have shown that IL-7 can enhance anti-tumour efficacy by promoting lymphocyte infiltration and improving the tumour microenvironment. Whether used as monotherapy or in combination with tumour vaccines, oncolytic viruses, or CAR T cell therapy, IL-7 enhances T cell infiltration, thereby improving anti-tumour efficacy. In addition, some studies have shown that not only does IL-7 not induce Treg proliferation, but it also reduces Treg levels. This modulation to improve or reverse the state of tumour immunosuppression and enhance the body’s immune response to tumours should be the focus and hotspot of current tumour research. IL-7 has a promising application in tumour immunotherapy because of its antagonistic effect on the state of immunosuppression.

CAR-T cells have achieved remarkable results in the treatment of various cancers, but some challenges remain. Although patients treated with CAR-T cells may achieve initial responses, many patients still experience frequent relapses and do not achieve complete eradication. Therefore, improving the proliferation and cytotoxicity of CAR-T cells, reducing depletion, and increasing infiltration remain pressing issues that need to be addressed urgently. IL-7 promotes the proliferation as well as increases the toxicity of CAR-T, and therefore, IL-7 with CAR-T cells may improve their efficacy against solid tumours and hold the promise of achieving the complete eradication of tumour cells.

The role of IL-7 in cancer cachexia continues to be a topic of great interest in the field of oncology. Further study is needed to fully elucidate the role of IL-7 in cancer cachexia and to determine whether targeting the IL-7 signalling pathway is a viable therapeutic strategy for mitigating the devastating effects of cachexia in cancer patients. In the meantime, a combination of immunotherapy and other therapeutic agents may be able to reverse the malignant process to some extent while improving the quality of life and survival of cancer patients. Some studies have also suggested that IL-7-based therapy may improve the prognosis of patients, but this idea still needs to be evaluated in more in-depth studies in the future [132]. The tumour microenvironment is a crucial factor in the development, progression, and prognosis of lung adenocarcinoma. It is worth noting that IL-7 inhibits tumour growth by regulating the proportion of immune iso-cell infiltration in the tumour immune microenvironment. Therefore, IL-7 can be used as a beneficial prognostic marker with the potential to be applied in the treatment of lung adenocarcinoma patients.

## Figures and Tables

**Table 2:** Current clinical trials are studying the use of IL-7 in cancer immunotherapy.

Cure	Combination	Cancers	Phases	NCT
IL-7		Bladder Acute Myeloid Leukaemia	I	NCT04054752
IL-7	Atezolizumab	Bladder Urothelial Carcinoma	II	NCT03513952
NT-I7		CESC	I	NCT04588038
	Pembrolizumab	Solid Tumours	I/II	NCT04332653
	Atezolizumab	NLCSC	II	NCT04594811
	Nivolumab	Gastro-Oesophageal Junction	I	NCT04594811
	Placebo	GBM	I	NCT02659800
	Placebo + Temozolomide + Radiation therapy	GBM	I/II	NCT03687957
	Atezolizumab	Skin Cancers	I/II	NCT03901573
IL-7-expressing CAR-T cells	CCL19	Advanced Malignant Solid Tumour	I	NCT03932565
	CCL19	Hepatocarcinoma	I	NCT03198546
	PD1 antibody	Lymphoma	I	NCT04381741
C7R-CAR-T	Cyclophosphamide	High-Grade Glioma	I	NCT04099797
	Cyclophosphamide + Fludarabine	Neuroblastoma Uveal Melanoma Breast Cancer	I	NCT03635632

**Table 3:** Presents clinical trials investigating cancer vaccines used in combination with IL-7 as an adjuvant for the treatment of the malignancy.

Vaccine	Trial ID	Phase	Conditions	Combination Adjuvant	Study Results	Reference
Sipuleucel-T	NCT01881867	II	Metastatic castration- resistant prostate cancer	None	Treatment with IL-7 was well tolerated. No improvements were observed in the IL-7 treatment group in terms of PFS or OS.	[98]
DC vaccination	NCT00923351	I/II	Paediatric sarcomas	None	No grade 3/4 AEs were reported. No difference in operating system (OS) was observed between subjects treated with or without IL-7.	[130]
MGN1601 vaccine	NCT00091338	I	Melanoma	Incomplete Freund’s adjuvant	NA	[131]

## Data Availability

Data are contained within the article.
